# Internal nasal dimensions of adults with nasal obstruction

**DOI:** 10.5935/1808-8694.20130103

**Published:** 2015-10-08

**Authors:** Inge Elly Kiemle Trindade, Priscila Capelato Prado Conegliam, Sergio Henrique Kiemle Trindade, Norimar Hernandes Dias, Ana Claudia Martins Sampaio-Teixeira

**Affiliations:** a Full Professor (Bauru School of Dentistry and Hospital for Rehabilitation of Craniofacial Anomalies of the University of São Paulo).; b MSc, Rehabilitation Sciences, Hospital for Rehabilitation of Craniofacial Anomalies of the University of São Paulo (Nurse, Hospital for Rehabilitation of Craniofacial Anomalies of the University of São Paulo).; c PhD, Otorhinolaryngology, School of Medicine of the University of São Paulo (MD, ENT, Botucatu School of Medicine and Bauru State Hospital).; d PhD, Otorhinolaryngology, Botucatu School of Medicine (MD, ENT, Botucatu School of Medicine and Bauru State Hospital).; e PhD, Sciences, Hospital for Rehabilitation of Craniofacial Anomalies of the University of São Paulo (Biologist, Physiology Laboratory, Hospital for Rehabilitation of Craniofacial Anomalies of the University of São Paulo).; Hospital for Rehabilitation of Craniofacial Anomalies/Dentistry School of Bauru, University of São Paulo.

**Keywords:** acoustic rhinometry, nasal cavity, nasal obstruction, nasal septum, sphenoidal conchae

## Abstract

Nasal septum deviation (SD) and turbinate hypertrophy (TH) increase the resistance to respiratory airflow and may impair nasal patency.

**Objective:**

To characterize the nasal geometry of individuals with nasal obstruction secondary to SD and/or TH by means of acoustic rhinometry.

**Method:**

This prospective study included 30 adults with complaints of nasal obstruction (NO) and SD + TH (n = 24), SD (n = 5) or TH (n = 1) seen by clinical examination. The cross-sectional areas of the three main dips of the rhinogram (CSA_1_, CSA_2_, CSA_3_), the distance between them and the nostrils (dCSA_1_, dCSA_2_, dCSA_3_), and the volumes of segments 1.0-3.2 cm (V_1_), 3.3-6.4 cm (V_2_), and 7.0-12.0 cm (V_3_) were measured before and after nasal decongestion (DN). For analysis, right and left cross-sectional areas and volumes were added and mean dCSA was calculated.

**Results:**

Mean values (± standard deviation) before ND were: 0.83 ± 0.23 (CSA_1_), 1.66 ± 0.52 (CSA_2_), and 2.36 ± 0.77 (CSA_3_) cm^2^; 2.19 ± 0.20 (dCSA_1_), 4.01 ± 0.33 (dCSA_2_), and 5.85 ± 0.37 (dCSA_3_) cm; 2.77 ± 0.51 (V_1_), 6.52 ± 1.99 (V_2_), and 26.00 ± 9.62 (V_3_) cm^3^; all values were lower than laboratory reference values (*p* < 0.05). ND led to proportionally greater increases of sectional areas and volumes in the NO group, suggesting an associated functional component. Individual analysis revealed 12 cases with normal results despite nasal obstruction.

**Conclusion:**

Most patients with structural nasal obstruction had results suggestive of nasal patency impairment in acoustic rhinometry.

## INTRODUCTION

The nose plays a series of vital functions. It filtrates, heats, and moistens inhaled air; it is the first line of defense against inhaled allergens; it acts as a sensory olfactory organ and affects resonance in speech production. Conditions such as deviated septum and turbinate hypertrophy affect nasal geometry and may impair nasal patency and the physiology of the nose, due to reductions in the inner dimensions of the nasal cavity and increases in the resistance to the flow of breathed air^1^, ^2^, ^3^.

Acoustic rhinometry can be used to verify nasal geometry in an objective non-invasive manner^4^, ^5^. This test uses the acoustic reflections of a sound pulse to measure the nose and the nasal cavity^6^.

This study aimed to measure the nasal cavities of adult patients with nasal obstruction caused by deviated septum and/or nasal concha hypertrophy and compare them to measurements made in subjects without signs of involvement. Cross-sectional areas, distances to the nostrils, and volumes of specific areas of the nasal cavity were analyzed and compared to reference values of individuals without signs of nasal obstruction described by Gomes^7^.

## METHOD

### The series

This study was approved by the Research Ethics Committee of the Craniofacial Rehabilitation Hospital of the University of São Paulo (HRAC/USP) and given permit 381/2006-SVAPEPE-CEP, and by the Research Committee of the Bauru State Hospital (HEB) as per permit HEB-CC-097/06.

This prospective study included an accidental sample of 30 adult individuals with nasal obstruction due to deviated septum and/or turbinate hypertrophy (Caucasian/brown adults of both genders, aged between 18 and 40 years). Participants were selected from a group of individuals who came to the ENT Clinic of the Bauru State Hospital (HEB) for nasal obstruction of any grade confirmed through physical examination at a later stage. Patients meeting the enrollment criteria were invited to join the study. Patients with enlarged pharyngeal tonsils, nasal obstruction of different etiologies such as previous nose surgery, or other conditions that prevented them from completing the study were excluded. Patients on nasal medication of any type were requested to stop treatment for five to seven days to undergo acoustic rhinometry.

### Clinical examination

**Figure 1:**
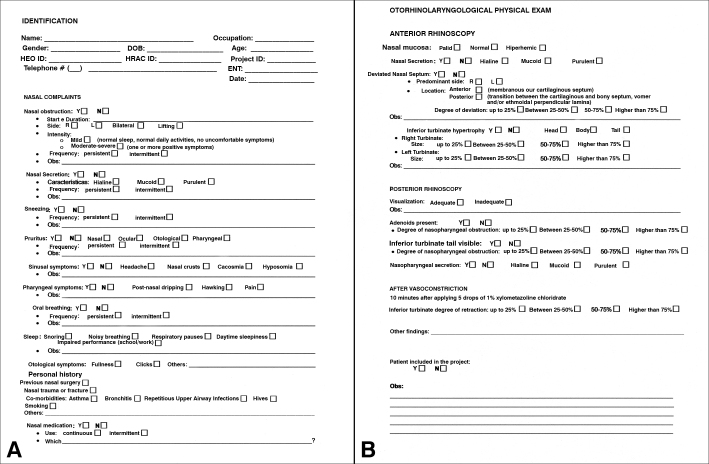
A: Interview protocol developed for this study; B: Physical ENT examination protocol developed for this study.

The diagnosis of nasal obstruction was based on the information collected during patient interviews and physical examinations performed in accordance with a protocol designed with this purpose (Figure 1A-B). The assessment protocol consisted primarily of a directed interview, in which the following data was collected:

1. time and duration of obstruction symptoms;
2. side of nasal obstruction;
3. frequency of nasal obstruction episodes;
4. rhinitis symptoms;
5. pharyngeal symptoms;
6. sinus symptoms;
7. ear symptoms;
8. nasal disease history;
9. associated diseases and habits.

Physical examination included anterior rhinoscopy, performed with the aid of a frontal light source and a nasal speculum, before and after administration of nasal vasoconstrictors, posterior rhinoscopy aided by a Garcia speculum to assess the rhinopharynx, otoscopy, and neck examination to capture possible associated lesions. When the more superior portions of the nasal fossae and rhinopharynx could not be assessed satisfactorily in clinical examination, subjects underwent examination with a Storz 3.4 mm endoscope. These measures were taken to rule out the presence of obstructive lesions in the respiratory portion of the nasal fossa such as tumors and inflammatory or neoplastic polyps. The characteristics of the nasal mucosa and nasal secretions, the degree and type of septum deviation, and the presence of inferior turbinate hypertrophy were analyzed during nasal cavity examination. The observed variables had a merely exploratory character and were considered only for the purposes of this study.

### Acoustic rhinometry

**Figure 2:**
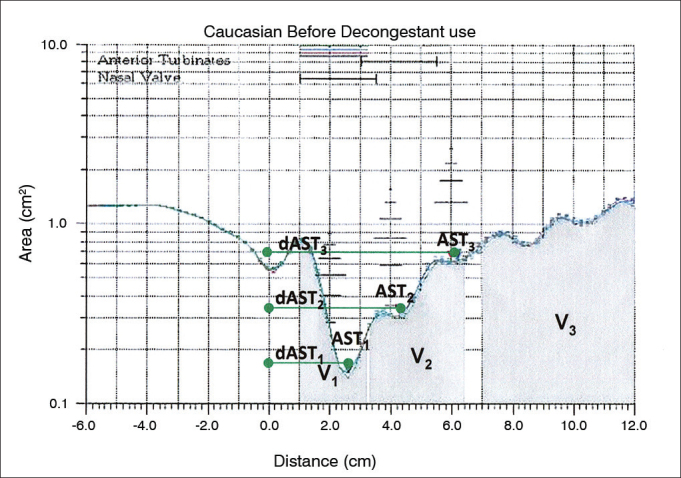
Typical rhinogram of a patient with nasal obstruction, showing the sites used to measure cross-sectional areas (CSA), distance from nostrils (dCSA), and nasal volumes (V).

Acoustic rhinometry was carried out at the Physiology Laboratory at HRAC/USP. An Eccovision Acoustic Rhinometer (HOOD Laboratories) was used and the tests were conducted as proposed by Trindade et al.^8^ and Gomes et al.^9^ Figure 2 shows a rhinogram from a patient with nasal obstruction.

The area-distance graph was used to calculate nasal cross-sectional areas (CSA) in square centimeters and the distance relative to the nostrils (dCSA) in centimeters in the rhinogram's second dip, corresponding to the area of the nasal valve (CSA_1_ and dCSA_1_), in the third dip (CSA_2_ and dCSA_2_), corresponding to the anterior end of the inferior and/or medial nasal concha, and in the fourth dip (CSA_3_ and dCSA_3_), corresponding to the medial-posterior end of the medial nasal concha^10^. The fist dip in the rhinogram, which corresponds to the area of the nostril, was not considered. For this reason, the three dips mentioned above were considered as the first, second, and third dips respectively. The integration of the area-distance curve was used to find the volumes in cubic centimeters^11^ of the segment situated between 10 and 32 mm from the nostril, matching the area of the nasal valve (V_1_), the segment situated between 33 and 64 mm from the nostril, corresponding to the nasal conchae (V_2_), and the segment situated between 70 and 120 mm from the nostrils, or the nasopharynx (V_3_). Areas and volumes were measured for the right and left nasal cavities and the results were combined. Examination was done before and 10 minutes after administering five drops of a nasal vasoconstrictor (0.1% xylometazoline hydrochloride).

### Data analysis

The variables were expressed as mean values ± standard deviation. The cross-sectional areas, distances from the nostrils, and nasal volumes seen in adult subjects with similar ages as the individuals in this study and without signs of nasal obstruction included in a previous study carried out at the Physiology Laboratory at the HRAC/USP^7^, ^8^, ^9^, ^12^ were used for comparison purposes. In order to meet the requirements of this study, the individual values verified for 60 nasal cavities of 30 patients without nasal obstruction were used to calculate the summation of the areas and volumes seen in the right and left nasal cavities of each individual and their mean distances. Table 1 shows the mean values seen for the group.

**Table 1:** Reference values for cross-sectional areas (CSA_1_, CSA_2_, CSA_3_), distances from nostrils (dCSA_1_, dCSA_2_, dCSA_3_), and volumes in adults without signs of nasal obstruction, before (Pre-ND) and after (Post-ND) the administration of nasal decongestants as reported by Gomes^7^ and Camargo^12^.

Variables	Pre-ND	Post-ND
CSA_1_ (cm^2^)	1.08 ± 0.21	1.13 ± 0.17 [+5%]
CSA_2_ (cm^2^)	1.95 ± 0.35	2.83 ± 0.40 [+45%]
CSA_3_ (cm^2^)	2.85 ± 0.51	3,97 ± 0.70 [+39%]
dCSA_1_ (cm)	2.12 ± 0.19	2.04 ± 0.17
dCSA_2_ (cm)	3.82 ± 0.34	3.89 ± 0.35
dCSA_3_ (cm)	5.74 ± 0.41	5.85 ± 0.51
V_1_ (cm^3^)	3.37 ± 0.50	3.65 ± 0.42 [+8%]
V_2_ (cm^3^)	7.95 ± 1.22	11.06 ± 1.70 [+39%]
V_3_ (cm^3^)	35.34 ± 7.13	45.41 ± 8.06 [+29%]

Acoustic rhinometry reference values minus two standard deviations were used to determine the lower limits of normality and help identify ranges of values suggestive of nasal obstruction^13^. *Student's t*-test was used to compare the groups (*p* < 0.05).

## RESULTS

For the purposes of analysis, the data observed for male and female subjects were combined into one group (n = 30), given that under baseline conditions, i.e., before the administration of nasal decongestants, no statistically significant differences were seen between genders. Clinical examination showed that 24 subjects had septum deviations associated with inferior nasal concha hypertrophy; five had septum deviations alone; and one had isolated inferior nasal concha hypertrophy.

Table 2 shows the mean values of cross-sectional areas and volumes (right and left sides combined) and distances of the three studied segments from the nostrils (mean right and left nostril distances) for patients with nasal obstruction.

**Table 2:** Cross-sectional areas (CSA_1_, CSA_2_, CSA_3_), distances from the nostrils (dCSA_1_, dCSA_2_, dCSA_3_), and nasal volumes of adults with nasal obstruction before (Pre-ND) and after (Post-ND) the administration of decongestants.

Variables	Pre-ND	Post-ND
CSA_1_ (cm^2^)	0.83 ± 0.23*	0.97 ± 0.20* [+17%]
CSA_2_ (cm^2^)	1.66 ± 0.52*	2.63 ± 0.57 [+58%]
CSA_3_ (cm^2^)	2.36 ± 0.77*	3.64 ± 0.76 [+54%]
dCSA_1_ (cm)	2.19 ± 0.20*	2.10 ± 0.26
dCSA_2_ (cm)	4.01 ± 0.33*	4.02 ± 0.30*
dCSA_3_ (cm)	5.85 ± 0.37	5.96 ± 0.49
V_1_ (cm^3^)	2.77 ± 0.51*	3.26 ± 0.46* [+18%]
V_2_ (cm^3^)	6.52 ± 1.99*	10.12 ± 1.80*[+55%]
V_3_ (cm^3^)	26.00 ± 9.62*	36.58 ± 8.66* [+41%]

mean percent variation in subjects with nasal obstruction after the administration of nasal decongestants;

* *p* < 0.05 statistically significant difference (nasal obstruction patients vs. reference values).

Comparisons against the values reported for controls (Table 1) revealed the CSA values of the group with nasal obstruction were significantly lower in the three considered segments before the administration of nasal decongestants. After decongestant administration, statistically significant differences were seen only in CSA_1_. Table 2 shows that percent variations in CSA_1_, CSA_2_, and CSA_3_ of patients with nasal obstruction after decongestant administration were +17%, +58%, and +54% in relation to individuals without nasal obstruction.

In regards to the distances from the nostrils of the analyzed segments, Table 2 shows that before the administration of nasal decongestants the mean dCSA_1_ and dCSA_2_ were significantly higher in the nasal obstruction group. After the administration of decongestants, statistically significant differences were seen only for dAST_2_.

Volumes before and after decongestants were significantly lower in the nasal obstruction group in all analyzed segments. The percent variation of the mean V_1_, V_2_, and V_3_ values after decongestants were +18%, +55%, and +41%, respectively, in relation to obstruction-free individuals.

The comparison of individual results against the range of normality calculated from the data published by Gomes^7^, together with the observation of the subjects' rhinograms, revealed that 18 patients in the nasal obstruction group had subnormal (below the limits of normality) cross-sectional areas in one or more of the analyzed segments (CSA_1_, CSA_2_, CSA_3_); the remaining patients (n = 12) had cross-sectional areas above the lower limits of normality.

The group with nasal obstruction was subdivided into two groups: nasal obstruction with subnormal CSA (n = 18) and nasal obstruction with normal CSA (n = 12). Mean cross-sectional areas were recalculated and compared to the reference mean values described by Gomes^7^, as shown in Table 3. The ‘nasal obstruction with normal CSA' subgroup was found to have significantly lower mean CSA_1_ values than the controls described by Gomes^7^. Subgroup ‘nasal obstruction with subnormal CSA' had statistically significant different values in the three analyzed segments when compared to the controls described by Gomes^7^ and to subgroup ‘nasal obstruction with normal CSA'.

**Table 3:** Comparison between nasal cross-sectional areas (CSA_1_, CSA_2_, CSA_3_) of adults without signs of nasal obstruction (C) described by Gomes^7^ and subgroups of adults with nasal obstruction and normal and subnormal CSA analyzed in this study, before (Pre-ND) and after (Post-ND) the administration of nasal decongestants. The data reflect the combination of values from the right and left sides.

Condition	Subgroup	CSA_1_ (cm^2^)	CSA_2_ (cm^2^)	CSA_3_ (cm^2^)
Pre-ND	Normal CSA (n = 12)	0.96 ± 0.19**	2.02 ± 0,41	2,83 ± 0,46
	Subnormal CSA (n = 18)	0.74 ± 0.21*# [+6%]	1.43 ± 0.46*#	2,04 ± 0,77*#

Post-ND	Normal CSA (n = 12)	1.02 ± 0.18* [+27%]	2.67 ± 0.40 [+32%]	3,76 ± 0,41 [+33%]
	Subnormal CSA (n = 18)	0.94 ± 0,21* [+27%]	2.61 ± 0.67 [+82%]	3,56 ± 0,92* [+74%]

* *p* < 0.05 statistically significant difference (nasal obstruction patients vs. reference values);

# *p* < 0.05 statistically significant difference (nasal obstruction patients with normal CSA vs. nasal obstruction patients with subnormal CSA).

Table 3 indicates that subgroup ‘nasal obstruction with subnormal CSA' had greater percent variations in CSA_1_, CSA_2_, and CSA_3_ (+27%, +82%, and +74%, respectively) than subgroup ‘nasal obstruction with normal CSA' (+6%, +32%, and +33%, respectively); the latter had cross-sectional areas comparable to the controls described by Gomes^7^.

## DISCUSSION

Three dips were seen in the baseline rhinograms of the subjects enrolled in this study, i.e., before the administration of decongestants, as previously described for normal adults and children^7^, ^8^, ^9^. The narrower segment, referred to herein as the minimum cross-sectional area (mCSA), was located in the anterior portion of the nasal cavity on 26 of the 30 subjects, within up to three centimeters of the nostrils, as described by Clement & Gordts^14^ in a paper published by the Standardization Committee on Objective Assessment of the Nasal Airway. Therefore, in these 26 cases CSA_1_ and mCSA were the same. The first dip in the rhinogram, which corresponds to the nasal valve and previously identified in European literature as the I-notch in reference to the *Isthmus nasi*, shifted in the anterior direction after the administration of decongestants, as observed in the original study by Grymer et al.^15^ Another dip was also seen in all rhinograms, situated ahead of the first dip and accounting for the nostrils. As it was deemed merely as a landmark (position 0.0), this dip was not considered in our analysis.

Comparison between results and reference values indicated that the mean cross-sectional areas of the three analyzed nasal segments - nasal valve (CSA_1_), anterior nasal concha (CSA_2_), and posterior nasal concha (CSA_3_) were significantly higher in the reference cross-sectional areas of adults without signs of nasal obstruction described by Gomes^7^. In that study, using a similar method, 60 nasal cavities were analyzed and found to have cross-sectional areas of 0.54, 0.98, and 1.42 cm^2^ respectively, while in this study the right-side cross-sectional areas were 0.45, 0.80, and 1.13 cm^2^, and the left-side cross-sectional areas were 0.37, 0.87 and 1.22 cm^2^ respectively. These values were significantly lower than the values observed for normal individuals, as also seen in the volumes of the three nasal segments - nasal valve (V_1_), nasal conchae (V_2_), and nasopharynx (V_3_), with mean volumes of 1.68, 3.98, and 18.93 cm^³^ in normal individuals, and 1.46, 3.17, and 13.19 cm^3^ on the right side and 1.31, 3.35, and 12.81 cm^³^ on the left side of individuals with nasal obstruction, respectively.

These values are similar to the values reported by various authors. However, comparisons require caution, as a number of uncontrolled variables present in these studies impact internal nasal measurements, such as degree of nasal obstruction, age, gender, posture, ethnic group, room temperature, external noise, and breathing, among others^6^, ^16^, ^17^, ^18^, ^19^, ^20^, ^21^. Individual data analysis suggested that most patients had some degree of involvement. Although the differences appear to be minimal, given the quadratic and cubic nature of the measurements, apparently small differences may actually indicate variations large enough to significantly alter nasal patency.

After topical administration of nasal decongestants, a procedure used routinely in acoustic rhinometry to minimize mucosal edema, all mean values were increased as also seen in normal adult populations^7^. The increase in mean cross-sectional areas and volumes observed after nasal decongestant administration was clearly greater in the nasal obstruction group than in what Gomes^7^ reported for normal subjects, indicating the presence of a functional component in the origin of the obstructive symptoms of the analyzed patients in addition to skeletal alterations seen in ENT examination. Symptoms consistent with nasal obstruction of a functional origin were reported in the interviews of some of the patients.

Although no differences were seen between nasal cavity sides, it is worth pointing out that the authors of this study considered the values resulting from the combination of both sides so as to avoid possible differences related to the nasal cycle. This is an episodic physiological phenomenon characterized by the alternation of mucosal congestion and decongestion cycles which reciprocally affect the nasal cavities, producing low-flow resting phases and high-flow active phases^22^, ^23^.

The mean distances from the nostril of the three dips in the rhinogram (dAST_1_, dAST_2_, dAST_3_) reported for adults in other studies^7^, ^12^ were 2.12, 3.82 and 5.74 cm, respectively. The group of subjects with nasal obstruction included in this study had mean distances from the right nostril of 2.14, 4.00 and 5.83 cm, respectively, and 2.24, 4.04 and 5.88 cm on the left side, respectively; higher values were seen for dAST_1_ and dAST_2_. Differences between sides did not appear to be clinically significant. The results observed in this study suggest that nasal obstruction may have caused sites of nasal constriction to move in the posterior direction, at least in part of the cases, a shift also seen after the removal of the impact of mucosal edema with the use of decongestants.

Visual analysis of rhinograms indicated that 12 of the 30 patients who had clinical evidences of nasal obstruction also had apparently normal rhinogram curves (ascending W-shaped curve). When the group was subdivided into two subgroups and the mean values were analyzed, it was found that subjects with subnormal areas (descending W-shaped curve) were different from control group subjects in all analyzed segments of the nose, while the group with 12 patients with paradoxically normal individual areas differed from the control group only in CSA_1_, possibly implying a trend toward small constrictions in areas of the nasal valve and explaining the subjective sensation of nasal obstruction experienced by these patients due to the resistive nature of this segment. More studies on this area are needed, as the sensation of obstruction is not limited to the size of the airway or by the ratio of laminar and turbulent flows, as suggested by Clement & Gordts^14^.

Although the subgroup of patients with subnormal values had clearly better response to therapy with decongestants than subjects with normal values, the 12 patients with normal values had limited response to decongestants when compared to normal subjects. Therefore, it should be investigated whether this behavior is not related to alterations in the autonomic regulation of the nasal mucosa, thus denoting diseased nasal mucosas unresponsive to adrenergic stimulation^24^.

In support of evidence-based medicine, this study has shown that acoustic rhinometry may contribute in the objective analysis of nasal airway obstruction, and add to the subjective data derived from scales and questionnaires and clinical ENT findings. In this aspect, this study also aided in the systematization of a clinical assessment protocol (Figure 1A-B) useful for the purposes of documentation and research^16^.

## CONCLUSION

This study revealed the presence of significant nasal patency involvement in patients with deviated septa and nasal concha hypertrophy confirmed through acoustic rhinometry. However, a subgroup of patients with normal acoustic rhinometry findings was identified in the group of patients with clinical signs of structural alterations; this group will be the topic of future studies.
